# Gender Differences in Recreational Sports Participation among Taiwanese Adults

**DOI:** 10.3390/ijerph120100829

**Published:** 2015-01-15

**Authors:** Liang-Ting Tsai, Feng-En Lo, Chih-Chien Yang, Joseph Jordan Keller, Shu-Yu Lyu

**Affiliations:** 1 Graduate Institute of Educational Information and Measurement, National Taichung University of Education, Taichung 40306, Taiwan; E-Mail: liangting.tsai@gmail.com; 2 Department of Leisure and Recreation Management, Asia University, Taichung 41354, Taiwan; E-Mail: lo.fengen@msa.hinet.net; 3 School of Public Health, College of Public Health and Nutrition, Taipei Medical University, Taipei 11031, Taiwan; E-Mails: joseph.jordan.keller@gmail.com (J.J.K.); sylyu@tmu.edu.tw (S.L.)

**Keywords:** recreational sports, Taiwan, motivation

## Abstract

This study examines the gender differences in the enjoyment of recreational sports participation among Taiwanese adults. Data were obtained using the 2007 Taiwan Social Change Survey. The questionnaire included a topical module of the International Social Survey Program regarding leisure time and sports. Results showed that male subjects were more likely to participate in recreational sports to improve their appearance and on account of their personal interest. In addition to these factors, female subjects also experienced greater motivation to participate when Taiwanese athletes performed well in international sporting competitions. This study confirmed that the factors influencing enjoyment of recreational sports participation differ among men and women. These results can be used to better inform public health professionals and other regulatory organizations formulating physical activity intervention strategies.

## 1. Introduction

Recreational sports are an important aspect of leisure activities that help relieve daily work stress. Recreational sports have a significant influence on human physiology and psychology. Physiologically, exercise has substantial benefits for promoting health and reducing mortality. Wen *et al.* highlighted that in Taiwan, regardless of gender or the existence of cardiovascular disease, people who exercise moderately (*i.e*., power walking) for an average of 15 min daily, or an accumulated 92 min per week, can experience up to a 14% decrease in mortality rate and can live an average of three years longer than those who do not exercise [1]. Jackson, Sui, Hébert, Church and Blair [2] and Sattelmair, Pertman and Forman [3] also reported that participation in recreational sports can reduce the occurrence of cardiovascular diseases.

From a psychological perspective, leisure activities involve social interaction. Interaction between social groups through leisure activities not only promotes physical and mental health, but can also reduce psychological pressure. Leisure activities can enhance self-esteem, thereby engendering a positive attitude and facilitating psychological development [4]. Participating in leisure activities can also prevent the development of depression [5]. Therefore, as recreational sports promote both physiological and psychological well-being, the promotion of recreational sports participation should be an important element in public health campaigns.

An individual’s motivation is a major factor influencing one’s participation in sports activities [6]. In psychology, motivation is commonly used to describe the aspect and strength of an individual’s active involvement efforts [4]. However, it is difficult to discern which factors should be targeted by public health campaigns seeking to motivate recreational sports participation, as previous studies investigating this relationship primarily concentrated on specific regions, age groups or sports [6,7,8,9,10]. Therefore, while the findings of these studies have provided a variety of significant contributions to recreational sports exploration and promotion, on account of their limited study regions, age groups and recreational sports participants, their findings cannot be generalized to other environments, people or athletic activities, thus seriously affecting the inference validity of these studies. 

Several related studies have demonstrated there to be substantial between-gender differences in recreational sports participation, with women reporting to be less interested in exercise and finding exercise less enjoyable than their male counterparts [4,7,10,11]. Tang [11] shows that there is a considerable relationship between gender and leisure activities. Girls are more inclined to static activities, while boys have a greater preference for dynamic activities. However, girls and boys are both reported to be interested in recreational sports participation for physical and mental health, personal interest and physical appearance. They also both enjoy participating in recreational activities [4,7]. Currently, research in Taiwan regarding public motivation for participating in recreational sports also remains limited to specific regions and specific groups.

Chuang [7] and James, Hsu, Redmond and Hope [12] reported that to increase exercise participation, exercise must be viewed as enjoyable and fun. Therefore, identifying the enjoyment that women obtain from exercise is important to increasing women’s participation in exercise. Therefore, as an analysis of the differences in recreational sports participation and motivation between genders in Taiwan remains lacking, we used the population of Taiwan as the sample for this investigation to explore the differences in behavioral factors and socioeconomic status that influence recreational sports participation and interests between genders.

## 2. Experimental Section

### 2.1. Sample and Data Collection

This study was based on a secondary data analysis of the results from the Taiwan Social Change Survey (TSCS) conducted by Academia Sinica in 2007 on a representative sample of the Taiwanese population. The data in this study were obtained from a representative sample of 2147 Taiwanese people who participated in the TSCS 2007, Phase 5, Wave 3. The TSCS was initiated in 1983 and is financially supported by the National Science Council. The main purpose of the survey is to provide survey data regarding various aspects of social change in Taiwan using national sampling [13]. The TSCS 2007 Leisure Time and Sports Questionnaire (LTSQ) design was based on social change trends in Taiwan and included the International Social Survey Program (ISSP) core questions, as well as topics regarding the current status of leisure life in Taiwan [14]. Specific Taiwanese citizens were selected for the questionnaire survey based on a three-stage stratified probability proportional to size design.

### 2.2. Measures

This study primarily investigated the factors that influence enjoyment of recreational sports participation. Recreational sports were defined as exercise-related activities that people choose to engage in during their spare or free time. The measurements used for analysis in this study are described below.

#### 2.2.1. Dependent Variable

This study employed the questionnaire item, “How much enjoyment do you attain from engaging in physical activities (e.g., exercising, going to the gym or walking)?” as the dependent variable. We then dichotomized the 5-point Likert scale responses coding “no enjoyment at all” and “not much enjoyment” as not enjoying exercise, and the responses “some enjoyment”, “very much enjoyment” and “extreme enjoyment” as enjoying exercise.

#### 2.2.2. Demographic Information

Respondents’ demographic characteristics included gender, age, degree of urbanization, educational level, marital status and occupational status.

#### 2.2.3. Behavioral Factors

Based on the findings of Chuang [7], Fu [4], Huang and Chen [8] and Phongsavan, McLean and Bauman [15], this study selected the following eight factors as independent variables: (1) frequency of participation in recreational sports; (2) to make friends; (3) for physical or mental health; (4) to meet other people; (5) to compete with others; (6) for physical appearance; (7) for individual interest; and (8) when Taiwanese athletes performed well in international sports or game competitions. Tang [11] indicated that there is a considerable relationship between gender and leisure activities. Girls are more inclined to static leisure activities, while boys more often prefer dynamic activities. However, girls and boys share common motivations for participation in recreational sports, including physical and mental health, personal interest and physical appearance. They also enjoy participating in recreational activities [4,7]. In recent years, researchers observed that the performance of Taiwanese athletes in international sports or game competitions leads more people to participate in and enjoy recreational sports. This is especially the case for baseball batting fields. Another purpose for participation in recreational sports is to help people meet others who share certain characteristics [8,16]. Thus, the variables, to make friends and to meet other people, were included in this study.

### 2.3. Statistical Analyses

A descriptive statistical analysis was conducted to provide the respondents’ demographic and other selected characteristics. A chi-square difference test was performed to assess the differences in respondent characteristics regarding the factors that may influence the enjoyment of recreational sports participation in Taiwan. Multivariable logistic regression models were used to test for associations between enjoyment of recreational sports participation and selected characteristics and factors. The multivariable logistic regression models were constructed separately for men and women: Model 1, all subjects (male and female); Model 2, only male subjects; and Model 3, only female subjects. The strength of association estimates was reported using odds ratios (OR) and 95% confidence intervals (CI) [17]. A *p*-value of less than 0.05 was considered statistically significant. To ensure that the study sample was representative of Taiwan’s population, data were analyzed with appropriate sampling weights, as included in the TSCS 2007 database.

## 3. Results

### 3.1. Differing Participant Characteristics Regarding Recreational Sports

The original survey sample size was 2147. However, as one of the participants did not respond to the item assessing the dependent variable, the final sample size of this study was 2146. The descriptive statistics are shown in Table 1. The overall participant population was 50.3% male and had an average age of 43.92 years (standard deviation = 16.41), ranging between 18 and 95 years of age. The average ages of the male and female participants were 44.23 years (standard deviation = 17.01) and 43.61 years (standard deviation = 15.78), respectively. The largest proportion of the participants (48.6%) lived in highly urbanized areas. Participants did not significantly differ in enjoyment of recreational sports participation by gender according to the chi-square test. However, the participants differed significantly in enjoyment of recreational sports participation according to their area of residence. Of the participants interested in recreational sports, over half (50.9%) lived in highly urbanized areas. The participants’ education levels were approximately evenly distributed. Furthermore, we found that the highest educational attainment of 56.1% of the study participants coded as not enjoying exercise was junior high school or below. The proportion of participants coded as not enjoying exercise decreased for each successive increase in education. Correspondingly, for recreational sports enjoyment, people with a junior college or above level of education accounted for the highest percentage (37.1%). The proportion of participants coded as enjoying exercise increased for each successive increase in education. Additionally, 62.9% of the participants were married, and 55.8% had full-time employment. The recreational sports enjoyment did not differ significantly by marital status or occupation.

**Table 1:** Participants’ characteristics and survey responses (*n* = 2146).

Variables	Enjoyment in Recreational Sports		Total *n* = 2146	*p*-value
	Yes	No		
	(*n* = 1653)	(*n* = 493)		
	*n* (%) = 77.1%	*n* (%) = 22.9%		
Gender				0.214
Women	809 (48.9)	257 (52.1)	1066 (49.7)	
Men	844 (51.1)	236 (47.9)	1080 (50.3)	--
Age group				0.012 ^*^
18–29	399 (24.2)	96 (19.5)	495 (23.1)	--
30–39	348 (21.1)	93 (18.9)	441 (20.6)	--
40–49	340 (20.6)	121 (24.5)	461 (21.5)	--
50–59	282 (17.1)	75 (15.2)	357 (16.6)	--
≥60	283 (17.1)	108 (21.9)	391 (18.2)	--
Degree of urbanization				<0.001 ^***^
High (core urban region/general urban region)	842 (50.9)	202 (41.0)	1044 (48.6)	--
Medium (emerging cities and townships/traditional industrial sector cities and townships)	577 (34.9)	168 (34.1)	745 (34.7)	--
Low (general townships and villages/rural areas)	234 (14.2)	123 (24.9)	357 (16.6)	--
Educational level				<0.001 ^***^
Junior high school or below	485 (29.3)	276 (56.1)	761 (35.5)	--
Senior high school	556 (33.6)	132 (26.8)	688 (32.1)	--
Junior college or above	613 (37.1)	84 (17.1)	697 (32.5)	--
Marital status				0.164
Single/divorced/widowed	627 (37.9)	170 (34.5)	797 (37.1)	--
Married	1026 (62.1)	323 (65.5)	1349 (62.9)	--
Occupational status				0.352
Others (students at school/unemployed/part-time job)	740 (44.8)	209 (42.4)	949 (44.2)	--
Full-time job	913 (55.2)	284 (57.6)	1197 (55.8)	--

^*^ *p* < 0.05; ^**^ *p* < 0.01; ^***^ *p* < 0.001.

### 3.2. Differing Independent Variables in Recreational Sports

Distribution of participants’ frequency characteristics among adults in Taiwan is shown in Table 2. Only 21.5% of the respondents participated in recreational sports daily. Among the respondents who enjoyed participating in recreational sports, only 26.5% engaged in recreational sports daily. In addition, 73.1% of the respondents were interested in making friends, and 78.6% of the respondents who enjoyed recreational sports were interested in meeting new friends. Of the respondents who enjoyed recreational sports, 88.5% believed that the purpose was to achieve physical health. Even among the respondents who did not enjoy recreational sports, 70.3% believed that the purpose of participating in recreational sports was physical health. Regardless of whether the respondents were interested in engaging in recreational sports, the vast majority of the participants did not believe that the purpose of participating in exercise was to compete with others, with 87.0% of those who did not enjoy recreational sports and 90.7% of those who did responding accordingly. Overall, 82.0% of the respondents felt that exercising because of an interest in exercise was important. Additionally, 94% of the respondents reported that they would feel proud if a Taiwanese athlete participated in an international competition and won an award, and 85.1% of the respondents who were not interested in sports would feel proud if a Taiwanese athlete participated in an international competition and won an award. To explore whether people had the same recreational sports enjoyment ratio under different behavioral factors, a chi-square test was conducted using a contingency table. Consequently, it was discovered that participants with differing behavioral factors showed significant differences regarding recreational sports enjoyment ratios and percentiles.

**Table 2:** Distribution of participants’ frequency characteristics among adults in Taiwan (*n* = 2146).

Variables	Enjoyment in Recreational Sports		Total *n* = 2146	*p*-value
	Yes	No		
	(*n* =1653)	(*n* = 493)		
	*n* (%) = 77.1%	*n* (%) = 22.9%		
Participation in recreational sports				<0.001 ^***^
Daily	438 (26.5)	23 (4.7)	461 (21.5)	--
Other (several times per month)	1215 (73.5)	470 (95.3)	1685 (78.5)	--
Make friends				<0.001 ^***^
Yes	1298 (78.6)	269 (54.7)	1567 (73.1)	--
No	353 (21.4)	223 (45.3)	576 (26.9)	--
Physical or mental health				<0.001 ^***^
Yes	1454 (88.5)	320 (70.3)	1774 (84.6)	--
No	189 (11.5)	135 (29.7)	324 (15.4)	--
Meet other people				<0.001 ^***^
Yes	868 (52.9)	190 (41.6)	1058 (50.4)	--
No	773 (47.1)	267 (58.4)	1040 (49.6)	--
Compete with others				0.033 ^*^
Yes	212 (13.0)	42 (9.3)	254 (12.2)	--
No	1420 (87.0)	410 (90.7)	1830 (87.8)	--
Physical appearance				<0.001 ^***^
Yes	783 (47.9)	139 (30.4)	922 (44.1)	--
No	850 (52.1)	318 (69.6)	1168 (55.9)	--
Individual interest				<0.001 ^***^
Yes	1344 (82.0)	299 (65.4)	1643 (78.4)	--
No	296 (18.0)	158 (34.6)	454 (21.6)	--
Taiwanese athletes performed well at international sports or game competitions				<0.001 ^***^
Yes	1512 (94.0)	383 (85.1)	1895 (92.1)	--
No	96 (6.0)	67 (14.9)	163 (7.9)	--

^*^ *p* < 0.05; ^**^ *p* < 0.01; ^***^ *p* < 0.001.

### 3.3. Gender Differences in Recreational Sports Participation

Multiple logistic regression analysis models were constructed to identify the statistically significant relationships between recreational sports participation and selected characteristics among all of the participants, the male participants alone and the female participants alone. Independent variables were selected based on their use in prior studies found in the literature [4,7,8,15]. We also computed a correlation matrix among all independent and dependent variables before proceeding with the multivariable logistic regression analysis. The minimum correlation coefficient is −0.490 for age and education level. The maximum correlation coefficient was −0.590 of 0.281 for to make friends and educational level. The results of the multivariate logistic regression are shown in Table 3. The factors associated with greater interest in recreational sports for all participants included: (1) male gender; (2) being aged 50 to 59 years; (3) medium or high urbanization of residence; (4) senior high school or junior college or above; (5) daily participation in recreational sports; (6) desire to make friends; (7) to improve health; (8) to enhance one’s personal appearance; (9) for personal interest; and (9) when Taiwanese athletes perform well at international sports competitions. The ORs of all variables can be seen in Table 3.

The factors related to increasing interest in recreational sports for the male participants included: (1) medium or high urbanization; (2) senior high school or junior college or above; (3) daily participation in recreational sports; (4) desire to make friends; (5) to improve health; (6) to enhance one’s personal appearance; and (7) for personal interest. The results showed that female participation in recreational sports is associated with: (1) being 30 to 39 years, 40 to 49 years or 50 to 59 years of age; (2) living in an area of medium or high urbanization; (3) having a senior high school or junior college or above level of education; (4) daily participation in recreational sports; (5) the desire to make friends; (6) to improve health; and (7) when Taiwanese athletes perform well at international sports competitions. The ORs also can be seen in Table 3.

**Table 3:** Multiple logistic regressions of the odds ratios with a 95% confidence interval for recreational sports among adults in Taiwan.

Variables	Model 1		Model 2 (Men)		Model 3 (Women)	
	OR (95% CI)	*p*	OR (95% CI)	*p*	OR (95% CI)	*p*
Gender						
Women	1.00	--	--	--	--	--
Men	1.30 (1.00–1.68)	0.046 ^*^	--	--	--	--
Age group						
18–29	1.00	--	--	--	--	--
30–39	1.33 (0.91–1.96)	0.142	0.77 (0.42–1.42)	0.409	2.07 (1.23–3.47)	0.006 ^**^
40–49	1.36 (0.92–2.01)	0.126	0.13 (0.47–1.53)	0.540	1.86 (1.08–3.17)	0.023 ^*^
50–59	2.36 (1.47–3.79)	<0.001 ^***^	1.37 (0.68–2.77)	0.377	3.28 (1.68–6.42)	<0.001 ^***^
≥60	1.64 (0.97–2.76)	0.065	1.39 (0.66–2.96)	0.386	1.78 (0.83–3.83)	0.138
Degree of urbanization						
Low	1.00	--	1.00	--	1.00	--
Medium	2.02 (1.45–2.82)	<0.001 ^***^	2.07 (1.27–3.36)	0.003 ^**^	2.00 (1.25–3.20)	0.004 ^**^
High	2.10 (1.49–2.97)	<0.001 ^***^	2.35 (1.42–3.88)	0.001 ^**^	1.91 (1.16–3.14)	0.010 ^*^
Education level						
Junior high school or below	1.00	--	1.00	--	1.00	--
Senior high school	2.87 (2.04–4.05)	<0.001 ^***^	4.30 (2.61–7.09)	<0.001 ^***^	1.96 (1.20–3.20)	0.007 ^**^
Junior college orabove	4.99 (3.38–7.36)	<0.001 ^***^	6.67 (3.75–11.88)	<0.001 ^***^	3.94 (2.30–6.77)	<0.001 ^***^
Recreational sports participation						
Other (several times per month)	1.00	--	1.00	--	1.00	--
Daily	9.16 (5.52–15.21)	<0.001 ^***^	7.63 (3.90–14.93)	<0.001 ^***^	12.09 (5.42–26.96)	<0.001 ^***^
Make friends						
No	1.00	--	1.00	--	1.00	--
Yes	2.26 (1.71–2.99)	<0.001 ^***^	2.10 (1.34–3.02)	0.001 ^**^	2.48 (1.67–3.67)	<0.001 ^***^
Physical or mental health						
No	1.00	--	1.00	--	1.00	--
Yes	2.09 (1.52–2.89)	<0.001 ^***^	2.38 (1.52–3.74)	<0.001 ^***^	1.90 (1.18–3.04)	0.008 ^**^
Meet other people						
No	1.00	--	1.00	--	1.00	--
Yes	1.01 (0.77–1.31)	0.98	0.91 (0.61–1.35)	0.648	1.11 (0.76–1.61)	0.588
Compete with others						
No	1.00	--	1.00	--	1.00	
Yes	1.08 (0.72–1.63)	0.716	1.13 (0.64–1.99)	0.686	0.88 (0.47–1.62)	0.680
Physical appearance						
No	1.00	--	1.00	--	1.00	--
Yes	1.14 (1.07–1.86)	0.014 ^*^	1.59 (1.03–2.46)	0.035 ^*^	1.27 (0.88–1.84)	0.205
Individual interest						
No	1.00	--	1.00	--	1.00	--
Yes	1.58 (1.18–2.12)	0.002 ^**^	1.92 (1.24–2.96)	0.003 ^**^	1.35 (0.903–2.03)	0.143
Taiwanese athletes performed well at international sports or game competitions						
Yes	1.00	--	1.00	--	1.00	--
No	1.80 (1.18–2.74)	0.006 ^**^	1.71 (0.95–3.07)	0.073	1.94 (1.05–3.59)	0.036 ^*^
Cox and Snell *R* square	0.178	--	0.205	--	0.167	--
Nagelkerke *R* square	0.280	--	0.327	--	0.261	--

^*^ *p* < 0.05; ^**^ *p* < 0.01; ^***^ *p* < 0.001; OR = odds ratio; CI = confidence interval.

## 4. Discussion and Conclusions

This study examined the between-gender differences in factors motivating recreational sports participation among adults in Taiwan. The results indicated a significant difference in recreational sports participation among men and women. This is likely due to the comprehensive nature of our study sample, which was a representative sample of all of Taiwan. The findings of this study have a high degree of consistency, reliability and effectiveness in the inference, that is they have a good degree of internal validity and external validity. For all participants, Taiwanese with a higher educational attainment, older age and more urbanized area of residence were more likely to be interested in recreational sports. Additional factors, such as the desire to make friends, improve physical or mental health and to enhance one’s physical appearance were also associated with the motivation to participate in recreational sports. The factors associated with greater motivation among men were physical appearance and personal interest. Among women, older age and the performance of Taiwanese athletes at international sports competitions were associated with a greater motivation to participate in recreational sports.

The contribution of recreational sports toward psychological and physical health has been well-recognized. Wendel-Vosetal [18] conducted a longitudinal analysis in the Netherlands, and the study found that regardless of gender, a positive correlation existed between increased recreational sports participation time and physical health. Pondè and Santana [19] highlighted that participating in recreational sports is essential for maintaining mental health among female laborers who have a low income or are dissatisfied with their employment. Thomsson [20] reported that Swedish women believe that participating in recreational sports benefits their appearance and promotes weight loss; thus, it is extremely suitable for women. The findings reported by Thomsson contradict those of this study conducted in Taiwan. Taiwanese women do not show increased interest in recreational sports to improve their appearance. Conversely, Taiwanese men tend to participate in recreational sports to improve their appearance.

Among women, older age was related to enjoyment of recreational sports participation. Federico *et al.* [20] assessed the extent that socioeconomic differences influenced physical activity and sports among Italian adults. Their results showed that age was positively associated with non-organized physical activity, yet negatively correlated with sports conducted within an organized context among both men and women [21]. 

The results of this study regarding Taiwanese adults differed quite significantly from those reported by Federico *et al.* [20]. In Taiwan, age did not affect the enjoyment of recreational sports participation among men. Furthermore, older female participants exhibited greater interest in recreational sports compared to their younger counterparts. The OR for the 50 to 59 years age group compared to the 18 to 29 years age group of women was 3.28. Considering this comparison, we believe that when Taiwanese women begin working or enter marriage, they have the primary responsibility of caring for the family, as well as working, which leaves them insufficient time to participate in leisure activities. As these women mature, they may gradually increase their participation in recreational sports, because of reduced family care responsibilities or changing health status. Therefore, men should be encouraged to help with family chores to increase women’s leisure time and reduce their family care responsibilities. This may help increase young women’s interest and participation in recreational sports. Family-style recreational sports, as opposed to the traditional segregation of men and women, can also be adopted to help encourage the participation of young women.

Compared to Taiwanese men, Taiwanese women’s interest in recreational sports participation was more easily influenced by external stimuli. This study found that Taiwanese women’s interest in recreational sports participation increased when Taiwanese athletes performed well at international sports competitions. In contrast, men’s participation in recreational sports did not differ significantly because of the scores of Taiwanese athletes in international competitions. For example, after the Taiwanese baseball team won third place in the 2001 Baseball World Cup, audiences for the 2002 Chinese Professional Baseball League games increased by 57.62%. Although more precise data revealed that this increase was mostly an increase in the number of women attending, the results confirm that it increased public willingness to participate in leisure activities. During the same period, the number of people visiting baseball batting fields also increased rapidly.

In this study, we explored the factors underlying the differences in enjoyment of recreational sports participation among Taiwanese men and women. This study conducted a broad spectrum exploration of the factors affecting the public’s interest in leisure activity participation. Future studies should explore the between-gender differences of various types of recreational sports to enhance the current understanding of the Taiwanese public’s enjoyment of recreational sports participation.
